# Transfection with GLS2 Glutaminase (GAB) Sensitizes Human Glioblastoma Cell Lines to Oxidative Stress by a Common Mechanism Involving Suppression of the PI3K/AKT Pathway

**DOI:** 10.3390/cancers11010115

**Published:** 2019-01-19

**Authors:** Ewelina Majewska, Javier Márquez, Jan Albrecht, Monika Szeliga

**Affiliations:** 1 Department of Neurotoxicology, Mossakowski Medical Research Centre, Polish Academy of Sciences, 5 Pawińskiego Street, 02-106 Warsaw, Poland; emajewska@imdik.pan.pl (E.M.); jalbrecht@imdik.pan.pl (J.A.); 2 Canceromics Laboratory, Department of Molecular Biology and Biochemistry, Faculty of Sciences, Campus de Teatinos, Instituto de Investigación Biomédica de Málaga (IBIMA), University of Málaga, 29071 Málaga, Spain; marquez@uma.es

**Keywords:** GLS2 glutaminase, human glioblastoma, PI3K/AKT signaling pathway, oxidative stress

## Abstract

*GLS*-encoded glutaminase promotes tumorigenesis, while *GLS2*-encoded glutaminase displays tumor-suppressive properties. In glioblastoma (GBM), the most aggressive brain tumor, *GLS* is highly expressed and in most cases *GLS2* is silenced. Previously, it was shown that transfection with a sequence encoding GAB, the main *GLS2* isoform, decreased the survival, growth, and ability to migrate of human GBM cells T98G and increased their sensitivity towards an alkylating agent temozolomide (TMZ) and oxidative stress compared to the controls, by a not well-defined mechanism. In this study we report that GAB transfection inhibits growth and increases susceptibility towards TMZ and H_2_O_2_-mediated oxidative stress of two other GBM cell lines, U87MG and LN229. We also show that in GAB-transfected cells treated with H_2_O_2_, the PI3K/AKT pathway is less induced compared to the pcDNA-transfected counterparts and that pretreatment with PDGF-BB, an activator of AKT, protects GAB-transfected cells from death caused by the H_2_O_2_ treatment. In conclusion, our results show that (i) GAB suppresses the malignant phenotype of the GBM cells of different tumorigenic potentials and genetic backgrounds and (ii) the GAB-mediated increase of sensitivity to oxidative stress is causally related to the inhibition of the PI3K/AKT pathway. The upregulation of the GLS2 expression and the inhibition of the PI3K/AKT pathway may become a novel combined therapeutic strategy for anti-glioma preclinical investigations.

## 1. Introduction

Glioblastoma (GBM, World Health Organization (WHO) IV grade) is the most common and highly lethal type of brain tumor. Despite aggressive treatment, GBM is still incurable, with a median survival time of 15 months. GBM is characterized by a high proliferation rate, infiltrative nature, and molecular heterogeneity [1].

Glutamine (Gln) plays a crucial role in tumor energetics and metabolism [2], and elevated catabolism of this amino acid is considered as a hallmark of malignancy [3]. Phosphate-activated glutaminase (GA, EC 3.5.1.2) converts Gln to glutamate (Glu) and ammonia. There are two genes coding for human GA: the *GLS* gene encodes GLS (kidney-type) isoforms, KGA, and GAC, and the *GLS2* gene codes for GLS2 (liver-type) isoforms, GAB and LGA [4,5,6].

Deregulated expression and/or activity of GA isoforms is a characteristic feature of neoplastic cell lines and tumors of different origins [7]. Growing evidence points to the opposing roles of GA isoforms in tumorigenesis. GLS isoforms are upregulated in highly proliferating cells, whereas the expression of GLS2 isoforms is related to resting or quiescent cell states [8]. The *GLS* gene is regulated by the mediators of oncogenesis such as MYC via miR-23s [9], Rho GTPases (Cdc42, Rac1, RhoC) [10], and Notch [11], while the *GLS2* gene was identified as a p53 tumor suppressor downstream target [12]. The diminishing expression or activity of GLS isoforms significantly decreased the proliferation of the prostate cancer cells [9], leukemic cells [13], Ehrlich ascites tumor cells [14], breast cancer cells [10,15], and glioblastoma cells [11,16]. A similar reversal of the phenotype was attained by the overexpression of *GLS2* in hepatocellular carcinoma (HCC) cells [12,17,18]. Moreover, the contribution of GLS2 to the antioxidant defense by the modulation of glutathione (GSH) and intracellular reactive oxygen species (ROS) levels has been documented in liver tumors [12,18].

*GLS2* in an overwhelming majority of GBM and GBM-derived cell lines is silenced [16,19,20] largely due to hypermethylation of the promoter [20]. Our previous research showed that stable transfection of human GBM T98G cell lines with a GAB cDNA sequence suppressed the malignant phenotype of these cells and altered the expression level of different genes encoding the proteins implicated in tumorigenesis [21]. Moreover, T98G cells transfected with GAB are more sensitive to oxidative agents, including hydrogen peroxide (H_2_O_2_) compared to their wild-type counterparts [22]. The question arose as to whether ectopic GAB expression results in similar phenotypical changes in other GBM cell lines displaying different genetic backgrounds. In this study, we examined the influence of GAB transfection on growth, the ability to migrate, and the sensitivity to H_2_O_2_ of two commercially available GBM cell lines, U87MG and LN229, varying with respect to *TP53* and *PTEN* status and tumorigenic potential. Next, we tested the hypothesis that GAB increases the sensitivity of GBM cells to H_2_O_2_ by a mechanism encompassing the downregulation of the phosphatidylinositol-3 kinase/protein kinase B (PI3K/AKT) cascade. This hypothesis was generated based on the following data: (i) H_2_O_2_ treatment enhances the phosphorylation of AKT in GBM cells [23]; (ii) the PI3K/AKT signaling pathway is associated with GBM development and the deregulation of elements related to this cascade results in uncontrolled tumor growth [24,25]; PI3K inhibitors are currently in clinical trials as anti-glioblastoma therapeutics [26]; and (iii) GAB decreases the phosphorylation level of AKT in HCC cells transfected with *GLS2* [17]. Here, we show that (i) transfection with GAB inhibits the growth of GBM cells and sensitizes them to H_2_O_2_ in three cell lines of different genetic backgrounds and (ii) increased sensitivity to H_2_O_2_ of all three GAB-transfected cell lines is related to the downregulation of the PI3K/AKT pathway.

## 2. Results

### 2.1. Stable Transfection of U87MG and LN229 Cells with GAB

**Figure 1:**
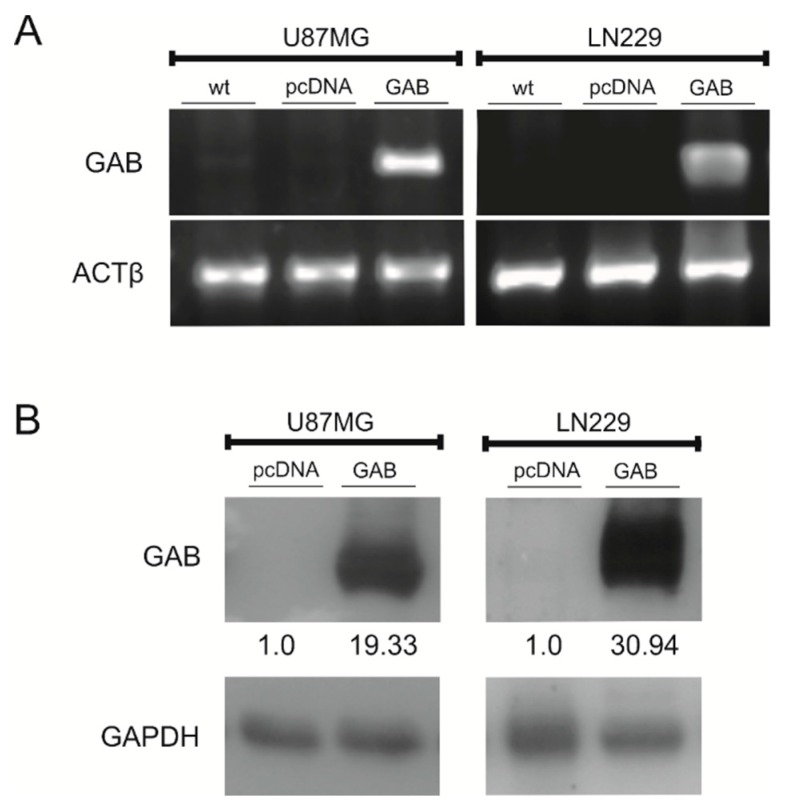
Analysis of the GAB levels in U87MG and LN229 cells wild-type (wt) or stably transfected with an empty pcDNA3 vector (pcDNA) or a pcDNA3 vector carrying GAB sequence (GAB). (**A**) GAB and ACTβ transcripts were determined by RT-PCR. (**B**) Protein levels of GAB and GAPDH in whole-cell lysates were determined by a Western blot analysis. A rabbit anti-GLS2 antibody detecting both isoforms arising from the *GLS2* gene was used.

Our previous study showed that transfection with cDNA encoding GAB reduced the viability, proliferation, and ability to migrate of T98G human GBM cells [21]. In order to examine the influence of the GAB transfection on the phenotype of other human GBM cell lines displaying different genetic background and tumorigenic potential than T98G cells, we first stably transfected U87MG and LN229 with a construct carrying the full human GAB sequence or empty pcDNA3 vector. While previously used T98G cells are mutant for *TP53* and *PTEN*, U87MG cells are wild-type for *TP53* and mutant for *PTEN*; in turn, LN229 cells are mutated for *TP53* and the wild-type for *PTEN*. Additionally, while T98G cells lack the ability to form tumors in nude mice, two others cell lines are considered as highly tumorigenic [27]. Wild-type (wt) and transfected with an empty pcDNA3 vector (−pcDNA) U87MG and LN229 cells do not express *GLS2* (Figure 1). GAB-transfected (−GAB) cells contain substantial amounts of GAB mRNA and protein (Figure 1). The expression of the GAB isoform was also confirmed in the GAB-transfected T98G cells (Figure S1).

### 2.2. Transfection with GAB Reduces Viability, Proliferation, and Ability to Migrate of U87MG and LN229 Cells and Sensitizes Them to Temozolomide (TMZ)

**Figure 2:**
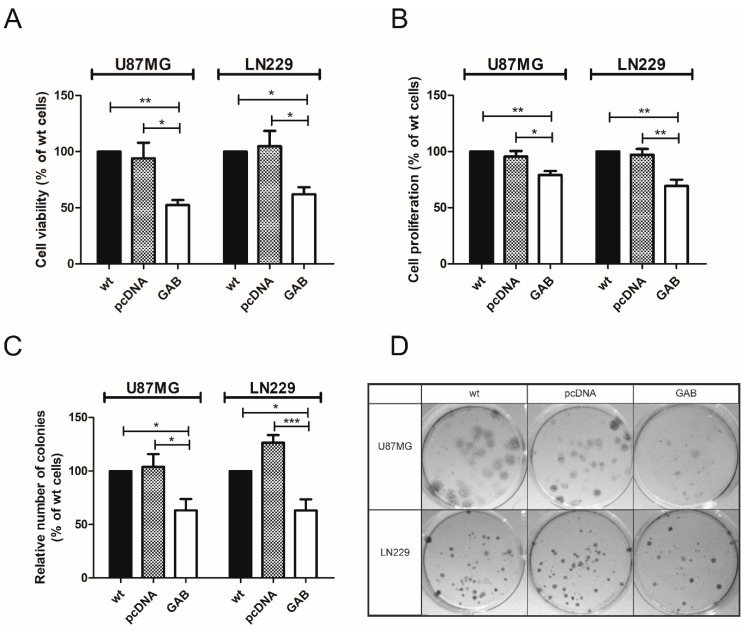
Transfection with the GAB sequence diminishes the viability, proliferation, and ability to form colonies of U87MG and LN229 cells. (**A**) Mitochondrial activity of wild type (wt) cells or cells stably transfected with the indicated plasmids was assessed by the MTT test 48 h after seeding. Results are mean ± SD (*n* = 4) expressed as a percentage of wt cells. * *p* < 0.05, ** *p* < 0.01 versus wt and pcDNA cells (one-way ANOVA followed by Tukey’s test). (**B**) Cell proliferation of wild type (wt) cells or cells stably transfected with the indicated plasmids was assessed by the BrdU assay 48 h after seeding. Results are mean ± SD (*n* = 4) expressed as a percentage of wt cells. * *p* < 0.05, ** *p* < 0.01 versus wt and pcDNA (one-way ANOVA followed by Tukey’s test). (**C**) Colony formation 3 weeks after seeding: the number of colonies are represented as a percentage relative to the number of colonies formed by wt cells. Results are mean ± SD (*n* = 5–7). * *p* < 0.05, *** *p* < 0.001 versus wt and pcDNA cells (one-way ANOVA followed by Tukey’s test). (**D**) Representative images of the plates with colonies formed within 3 weeks of growth.

The influence of GAB transfection on cell viability was assessed by a MTT assay. UGAB cells showed a 48% decrease in viability as compared to the controls (U87MG and UpcDNA). The viability of the LNGAB cells was reduced by 38% as compared to the controls (LN229 and LNpcDNA) (Figure 2A).

Next, we analyzed the growth of GAB-transfected cells by performing proliferation and clonogenic assays. The UGAB cells exhibited a reduction in proliferation rate by 21% as compared to the controls. The LNGAB cells presented a 31% decrease in proliferation rate as compared to the controls (Figure 2B). Both the UGAB and LNGAB cells showed significantly lower colony formation rates as compared to the controls (Figure 2C,D).

**Figure 3:**
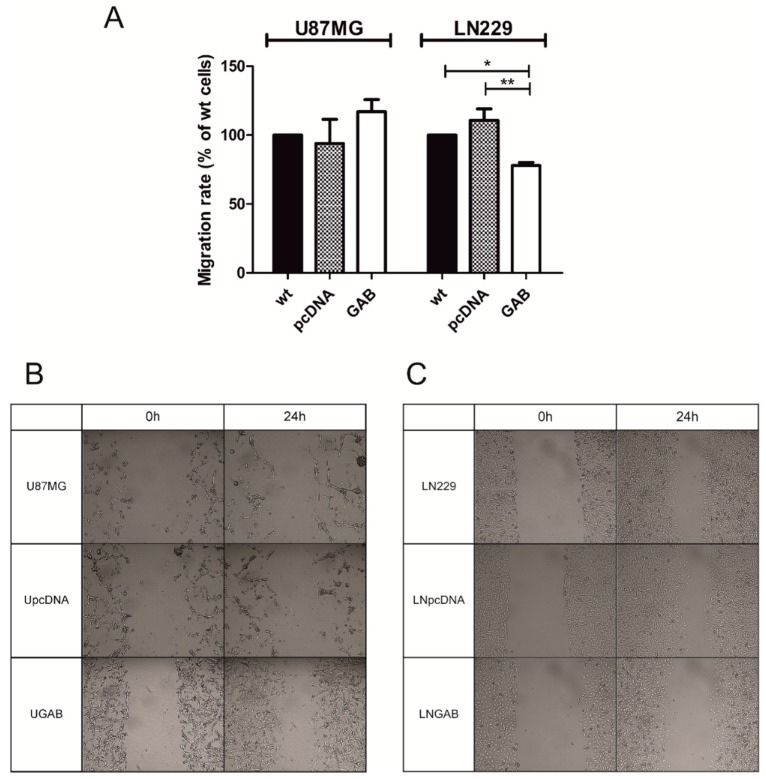
The influence of the transfection with the GAB sequence on the migration ability of U87MG and LN229 cells. (**A**) The migration rate of wt cells or cells stably transfected with the indicated plasmids was measured by the wound-healing. Twenty-four hours after seeding, the confluent cells were scratch-wounded with a micropipette tip. Wound borders were recorded and measured at 0 h and 24 h post-scratching. Results are mean ± SD (*n* = 4–5) expressed as a percentage of the scratch gap observed for wt cells. *p < 0.05, ** *p* < 0.01 versus wt and pcDNA cells (one-way ANOVA followed by Tukey’s test). (**B**,**C**) Representative images of the scratch gaps taken at 0 h and 24 h after scratching. Magnification: objective 4× and digital 10×.

To investigate the effect of GAB transfection on the cells migration we used a wound-healing assay. While no changes in the ability to migrate were observed in the UGAB cells as compared to the controls (Figure 3A,B), the LNGAB cells exhibited a 22% inhibition of migration compared to the controls (Figure 3A,C). Consistent with a previous study [21], we observed a significant reduction of the viability, proliferation, and ability to form colonies and to migrate in T98G cells upon transfection with the GAB sequence (Figure S2A–F).

**Figure 4:**
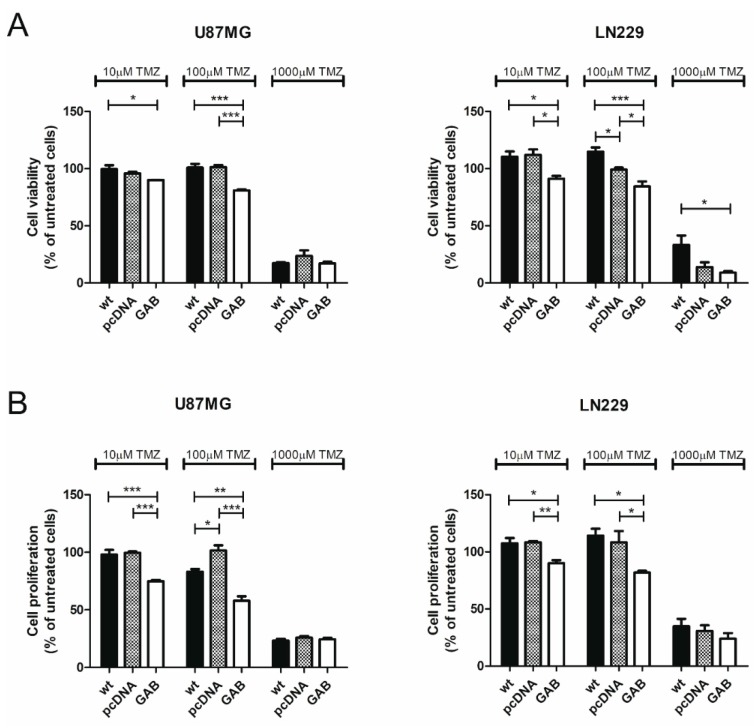
Transfection with the GAB sequence sensitizes U87MG and LN229 cells to temozolomide (TMZ) and diminishes viability and proliferation. (**A**) Wild type (wt) cells or cells stably transfected with the indicated plasmids (U87MG cells left panel and LN229 cells right panel) were exposed to increasing concentrations of TMZ for 72 h, and then mitochondrial activity was assessed by the MTT test. Results are mean ± SD (*n* = 4) expressed as a percentage of untreated cells. * *p* < 0.05, *** *p* < 0.001 versus wt and pcDNA cells (one-way ANOVA followed by Tukey’s test). (**B**) Cell proliferation of wild type (wt) cells or cells stably transfected with the indicated plasmids (U87MG cells left panel and LN229 cells right panel) were exposed to increasing concentrations of TMZ for 72 h, and then cell proliferation was assessed by the BrdU assay. Results are mean ± SD (*n* = 4) expressed as a percentage of wt cells. * *p* < 0.05, ** *p* < 0.01, *** *p* < 0.001 versus wt and pcDNA (one-way ANOVA followed by Tukey’s test).

Our previous study showed that transfection with GAB sensitized T98G cells to treatment with TMZ, an alkylating agent often used in GBM therapy [28]. A similar effect was observed in U87MG and LN229 cells. In both cell lines GAB-transfected cells turned out to be significantly more sensitive to treatment with TMZ in viability and proliferation assays compared to the controls (Figure 4).

### 2.3. Transfection with GAB Sensitizes U87MG and LN229 Cells to H_2_O_2_ Treatment

**Figure 5:**
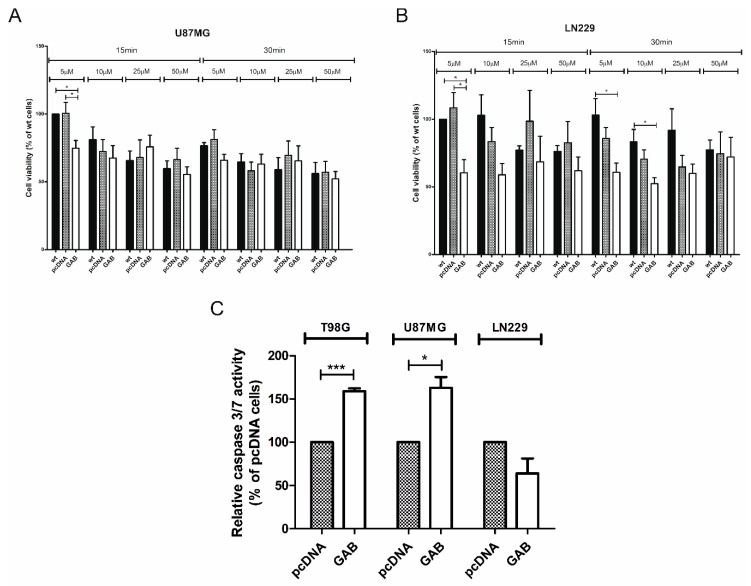
Transfection with the GAB sequence alters the sensitivity of cells to H_2_O_2_, inducing in some cell lines apoptosis. The sensitization of U87MG (**A**) and LN229 (**B**) cells to treatment with H_2_O_2_ are shown here. The viability of wt cells or transfected with the indicated plasmids was measured by the MTT test after treatment with 5–50 μM H_2_O_2_ for 15 min and 30 min. Results are mean ± SD (*n* = 3–5) expressed as a percentage of wt cells treated with 5 μM H_2_O_2_ for 15 min. * *p* < 0.05 versus wt and pcDNA (one-way ANOVA followed by Tukey’s test). (**C**) Transfection with GAB upon H_2_O_2_ treatment induces apoptosis in T98G and U87MG cells. Apoptosis was analyzed by caspase 3/7 activity analysis in (i) TpcDNA and TGAB cells after the treatment with 200 µM H_2_O_2_ for 15 min, (ii) UpcDNA and UGAB cells after the treatment with 5 µM H_2_O_2_ for 15 min, and (iii) LNpcDNA and LNGAB cells after the treatment with 5 µM H_2_O_2_ for 15 min. Caspase-Glo 3/7 assay results are shown as a percentage of pcDNA cells. Results are mean ± SD (*n* = 3–4) expressed as a percentage of pcDNA cells. * *p* < 0.05, *** *p* < 0.001 versus pcDNA (Student’s *t*-test).

Previous research showed that transfection with GAB sensitizes T98G cells to H_2_O_2_ treatment: A significant dose-dependent decrease in TGAB cells viability was observed when the cells were treated with 200 µM H_2_O_2_ for 15 and 60 min as well as 300 µM H_2_O_2_ for 60 min [22]. In this study we observed a decreased viability of TGAB cells at 50, 100, 200, and 300 µM H_2_O_2_ added for 15 and 60 min (Figure S2G). To assess the influence of GAB transfection on the sensitivity of U87MG and LN229 cells to the lower concentrations of H_2_O_2_ (5, 10, 25, and 50 µM), shorter treatment times (15 and 30 min) had to be applied. In most of the treatment conditions, both UGAB and LNGAB cells appeared to be more sensitive to H_2_O_2_ compared to the controls. A statistically significant decrease in the viability of GAB transfectants was observed when 5 µM H_2_O_2_ treatment for 15 min was applied (Figure 5A,B). Therefore, these conditions were used in further experiments.

### 2.4. Transfection with GAB Induces Caspase 3/7 Activity in TGAB and UGAB Cells

According to the literature data, treatment with H_2_O_2_ triggers apoptosis in GBM cells [23]. Therefore, we analyzed the activity of caspase 3/7, effectors of apoptosis, in pcDNA- or GAB-transfected cells treated with H_2_O_2_. Upon H_2_O_2_ treatment, TGAB and UGAB cells presented increased caspase 3/7 activity as compared to their pcDNA-transfected counterparts (Figure 5C). Of note, the lack of change in this parameter was observed between the LNGAB and LNpcDNA cells. (Figure 5C).

### 2.5. Transfection with GAB Suppresses pAKT Signaling Pathway

**Figure 6:**
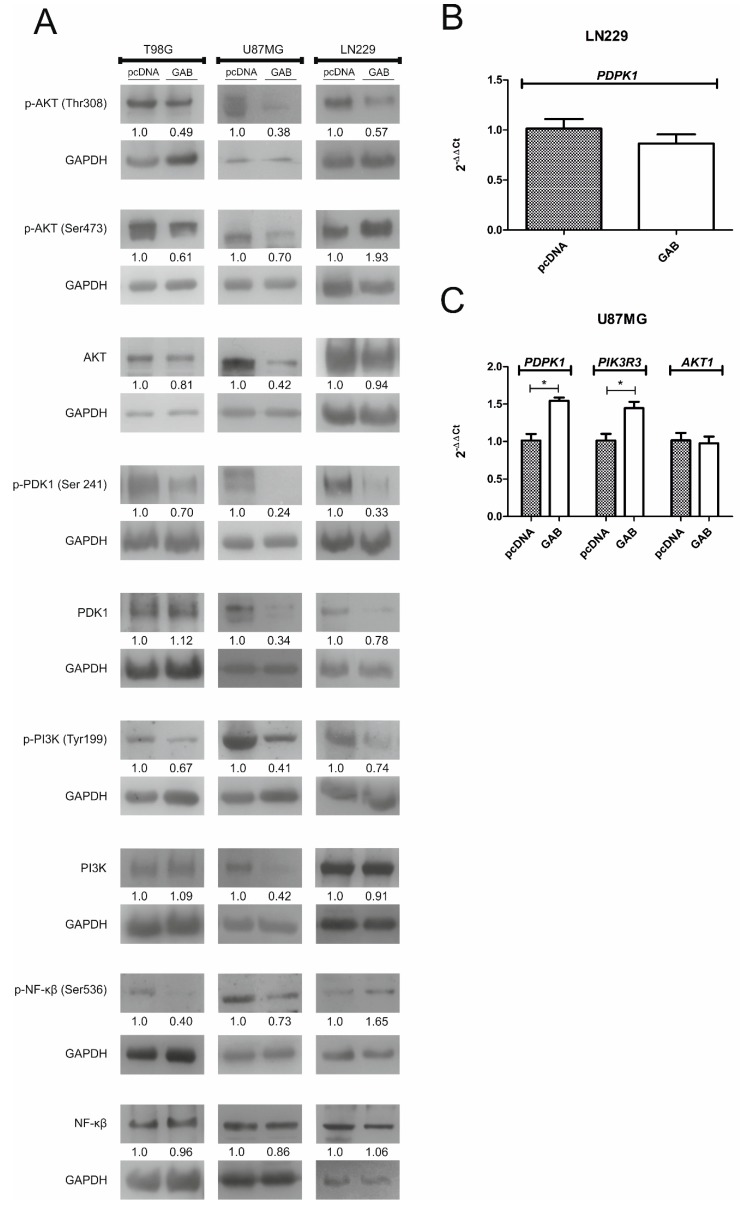
Transfection with the GAB sequence alters the level of proteins related to the PI3K/AKT signaling pathway upon H_2_O_2_ treatment. (**A**) The level of proteins were analyzed by the Western blot analysis in (i) TpcDNA and TGAB cells after the treatment with 300 µM H_2_O_2_ for 1 h, (ii) UpcDNA and UGAB cells after the treatment with 5 µM H_2_O_2_ for 15 min, and (iii) LNpcDNA and LNGAB cells after the treatment with 5 µM H_2_O_2_ for 15 min. Results are mean ± SD (*n* = 3–5) expressed as a percentage of the bands density observed for pcDNA cells. (**B**) The expression of the mRNAs coding for PDK1 in LNGAB and LNpcDNA cells are shown. Results are mean ± SD (*n* = 4). (**C**) The expression of the mRNAs coding for PDK1, PI3K, and AKT in UGAB and UpcDNA cells are shown. Results are mean ± SD (*n* = 4). * *p* < 0.05 versus pcDNA (Student’s *t*-test).

We next analyzed the levels of molecules belonging to the PI3K/AKT pathway in –pcDNA and –GAB transfected cells treated with H_2_O_2_ (Figure 6). All GAB-transfected cell lines showed a decreased phosphorylation level of AKT on Thr308 as compared to the controls. The TGAB and UGAB cells exhibited also a diminished AKT phosphorylation level on Ser473, in contrast to the LNGAB cells, in which an increase was observed. While UGAB cells presented a significant decrease in a total AKT level as compared to the pcDNA transfected counterparts, the lack of changes in the AKT level was observed between the TGAB and TpcDNA and the LNGAB and LNpcDNA cells, respectively. The level of pPDK1 and pPI3K, proteins which are involved in AKT phosphorylation on Thr308, was decreased in all the GAB-transfected cells compared to the controls. The total PDK1 protein level was lowered in the UGAB and LNGAB cells whereas the PI3K level was diminished only in U87GAB cells as compared to the controls. The TGAB and UGAB cell lines showed a diminished phosphorylation level of NF-κB with no alterations in a total protein level as compared to the controls (Figure 6A).

The changes in the levels of total PDK1, PI3K, and AKT observed in the U87MG set and LN229 set prompted us to analyze the expression of the genes coding for these proteins. While we did not find any difference in the level of *PDK1* transcript between the LNpcDNA and LNGAB cells treated with H_2_O_2_ (Figure 6B), a significant increase in the level of this mRNA was observed in the UGAB cells compared to the UpcDNA cells (Figure 6C). Moreover, UGAB cells treated with H_2_O_2_ displayed an increased level of *PI3K* transcript compared to UpcDNA cells (Figure 6C). No difference in *AKT* mRNA level (encoded by *AKT1* gene) was found between UGAB and UpcDNA cells treated with H_2_O_2_ (Figure 6C).

### 2.6. GAB-Evoked Downregulation of pAKT Pathway Contributes to Increased Sensitivity to H_2_O_2_ Treatment

**Figure 7:**
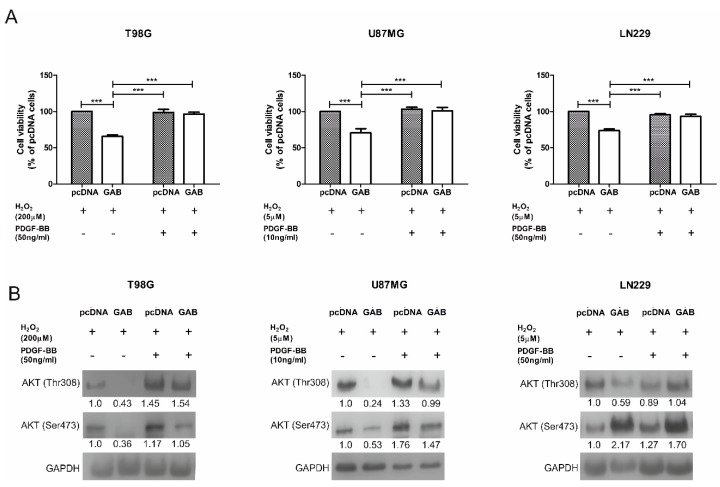
Pretreatment with PDGF-BB, an AKT pathway activator, restores the resistance to H_2_O_2_ treatment. (**A**) (i) The viability of T98G cells transfected with an empty pcDNA or carrying the GAB sequence vector cultured in media with or without PDGF-BB (50 ng/mL) for 24 h before exposure to H_2_O_2_ (200 µM, 15 min); (ii) the viability of U87MG cells transfected with an empty pcDNA or carrying the GAB sequence vector cultured in media with or without PDGF-BB (10 ng/mL) for 24 h before exposure to H_2_O_2_ (5 µM, 15 min); and (iii) the viability of LN229 cells transfected with an empty pcDNA or carrying the GAB sequence vector cultured in media with or without PDGF-BB (50 ng/mL) for 24 h before exposure to H_2_O_2_ (5 µM, 15 min). Cell viability was determined by an MTT assay, and the results are expressed as a percentage of cells transfected with an empty pcDNA vector non-pretreated with PDGF-BB. Results are mean ± SD (*n* = 4–5). *** *p* < 0.001 (one-way ANOVA followed by Tukey’s test). (**B**) An influence of PDGF-BB treatment on the level of AKT (Thr308) and AKT (Ser473) in T98G, U87MG, and LN229 cell lines was analyzed by a western blot. Results are mean ± SD (*n* = 3–4) expressed as a percentage of pcDNA cells.

Decreased levels of the key proteins involved in pAKT pathway observed in GAB-transfected cells treated with H_2_O_2_ are not direct proof that this phenomenon contributes to the increased sensitivity to H_2_O_2_. Therefore, in the next experiment, we pretreated −pcDNA and −GAB cells with PDGF-BB, an activator of AKT phosphorylation [29], for 24 h, and then the sensitivity to H_2_O_2_ was assessed as described above. The concentrations of PDGF-BB were chosen based on experiments described in the literature [30]. The H_2_O_2_ concentrations and time of treatment were selected based on experiments described above in which –GAB cells presented an increased susceptibility to H_2_O_2_. Cells treated with vehicles were used as a reference. Pretreatment with PDGF-BB resulted in an increased viability of all –GAB cell lines upon the H_2_O_2_ treatment as compared to the vehicle-treated counterparts. Pretreatment with PDGF-BB increased the viability of TGAB after the H_2_O_2_ treatment from 66–96%, the viability of UGAB cells from 70–101%, and the viability of LNGAB cells from 74–94% (Figure 7A). Pretreatment with PDGF-BB did not change the viability of –pcDNA cell lines treated with H_2_O_2_ (Figure 7A).

The analysis of the AKT phosphorylation level confirmed that pretreatment with PDGF-BB increased the Thr308 phosphorylation in all –GAB cells treated with H_2_O_2_ as compared to –GAB cells pretreated with the vehicle (Figure 7B). Pretreatment with PDGF-BB did not change the phosphorylation level on Thr308 in –pcDNA cells treated with H_2_O_2_ as compared to the vehicle treated –pcDNA cells. Pretreatment with PDGF-BB increased the phosphorylation level on Ser473 in TGAB cells treated with H_2_O_2_ and did not affect it in TpcDNA cells as compared to the vehicle treated cells. UGAB and UpcDNA cells presented a similar tendency to that observed in T98G transfectants. The level of phosphorylation on Ser473 in LNGAB pretreated with PDGF-BB or the vehicle was higher than that observed in LNpcDNA cells (Figure 7B).

## 3. Discussion

Our previous study demonstrated that transfection with the GLS2 isozyme GAB, a target of the p53 family of tumor suppressors, diminished the viability, proliferation, and ability to migrate of T98G cells [21]. Here we extended the analysis of the effects of the GAB transfection to U87MG and LN229, the GBM cell lines which differ from T98G cells and from each other with respect to their tumorigenic potential and the status of the tumor suppressor genes frequently mutated in GBM: *TP53* and *PTEN* [27]. Our data clearly demonstrates that exogenous GAB decreases the viability, growth, and proliferation of U87MG and LN229 cells, pointing to the ubiquity of this phenomenon among GBM cell lines originally not expressing *GLS2*, independently of the TP53/PTEN status of these highly malignant glioma cells. Therefore, the tumor-suppressive effect induced by GLS2 must also involve p53-independent mechanisms. Of note, we found a discrepancy in the effect of the GAB transfection on the ability to migrate between U87MG cells and two other cell lines. While reduced migration was observed in TGAB and LNGAB cells compared to the controls, no differences in this parameter were detected between the UGAB cells and the controls. The reasons of this discrepancy between the U87MG cells and two other cell lines are unclear. Ramao and coworkers provided evidence that U87MG cells displayed a higher basal migration rate compared to T98G cells [31]. Moreover, Esencay and coworkers observed a reduced migration of LN229 cells but not U87MG cells towards a stromal-derived factor (SDF)-1α in hypoxic conditions [32]. However, the detailed molecular mechanism underlying these discrepancies has not been proposed.

The earlier findings suggested that GAB overexpression potentiated the effect of TMZ and oxidative stress on the viability of T98G cells [22]. Here we confirmed these data and observed an increased sensitivity to TMZ and H_2_O_2_ of GAB-transfected cells in U87MG and LN229 cell lines. In order to shed some light on the mechanism contributing to this phenomenon, we measured the levels of molecules belonging to the PI3K/AKT pathway in pcDNA- and GAB-transfected cells treated with H_2_O_2_; GAB negatively regulates this pathway in hepatocellular carcinoma cells [17], and the induction of the phosphorylation of AKT was previously observed upon H_2_O_2_ treatment in T98G cells [23]. These data prompted us to hypothesize that in GAB-transfected GBM cells treated with H_2_O_2_; the PI3K/AKT pathway could be less induced than in pcDNA-transfected cells, and this phenomenon could lead to the increased sensitivity to H_2_O_2_. Indeed, we observed a significantly reduced AKT phosphorylation level on Thr308 upon H_2_O_2_ treatment in all three cell lines transfected with GAB compared to the pcDNA-transfected counterparts. Moreover, in these conditions, T98G and U87MG cells enriched in GAB presented lower levels of AKT phosphorylated on Ser473. Phosphorylation on Thr308 residue is both necessary and sufficient for AKT activation, although the maximal activation is acquired after additional phosphorylation on Ser473 (for review see Reference [25]). Therefore, we assume that in all three cell lines used in this study, exogenous GAB translates to the decreased induction of AKT, albeit different mechanisms are involved in this decrease. While no changes in the level of total AKT was found in T98G and LN229 cell sets, a significantly diminished level of this protein, but not AKT transcript, was observed in UGAB cells as compared to UpcDNA cells. These data suggest the modulation of AKT activity on a post-translational level in T98G and LN229 cell sets and on both translational and post-translational levels in U87MG cell set. The mechanism underlying decreased AKT phosphorylation in GAB-transfected cells of all three cell lines is most likely associated with the diminished levels of pPDK1, the kinase phosphorylating AKT on Thr308, and pPI3K, another modulator of AKT activity (for review see [25]) observed in these cells. However, in U87MG cells, GAB seems to affect also the translation of *PDK1* and *PI3K* mRNAs.

The precise molecular mechanism of the downregulation of the PI3K/AKT pathway by GAB remains unclear. The nuclear localization of GAB in the neurons and astrocytes and its interactions with PDZ-containing proteins suggest that the role of this protein may go beyond GA activity [33,34,35]. Increasing evidence suggests the relevance of interactions between several proteins and their PDZ domain containing partners in the regulation of the PI3K/AKT pathway [36,37]. Therefore, it is possible that GAB interacts with some upstream signal proteins of the PI3K/AKT pathway. On the other hand, our earlier study showed that GAB modified gene expression patterns [21]. Further studies are required to determine whether the transcription alterations observed upon transfection with GAB may modulate the PI3K/AKT cascade activity. Moreover, the influence of GAB on the downstream effectors of the PI3K/AKT pathway has to be elucidated. One of these effectors is NF-κB which is involved in carcinogenesis by the activation of the pro-survival and antiapoptotic genes [38]. In this study, TGAB and UGAB cells treated with H_2_O_2_ displayed a significant reduction of NF-κB phosphorylation and an increased activity of caspase 3 and 7 as compared to their pcDNA-transfected counterparts. These findings suggest that in T98G and U87MG cells exposed to H_2_O_2_, exogenous GAB promotes apoptosis which is most likely mediated by the downregulation of NF-κB activity, supporting the notion that GAB possesses proapoptotic properties [22]. Of note, the treatment of LNGAB cells with H_2_O_2_ tended to increase the level of phosphorylated NF-κB but did not change the activity of caspase 3 and 7, which implies that in this particular cell line, the mechanism underlying GAB-mediated cell death is other than caspase dependent apoptosis, e.g., autophagy or senescence. Further studies to identify this mechanism are under way in our laboratory. It is tempting to infer that the lack of proapoptotic effect of the GAB transfection in LN229 cells is mechanistically related to the increased phosphorylation of AKT at Ser473 residue, a response exactly opposite to that obtained on two other cell lines.

Regardless of the differences between distinct cell lines in the influence of exogenous GAB on the particular molecules belonging to the PI3K/AKT pathway, the decreased level of AKT phosphorylation in GAB-transfected cells compared to the controls is observed in all cell lines examined. Our results clearly indicate that the GAB-evoked downregulation of AKT phosphorylation contributes to the increased sensitivity of GBM cells towards H_2_O_2_. This conclusion is based on the finding that pretreatment with PDGF-BB, an activator of AKT [29], protects GAB-transfected cells from death caused by the H_2_O_2_ treatment. Our results support the previous notion that the negative regulation of PI3K/AKT signaling mediates GAB’s role in the suppression of hepatocellular carcinoma growth [17]. Moreover, our data are consistent with previous reports on the role of reactive oxygen species, like H_2_O_2_, on tumor cell survival mediated by the PI3K/AKT pathway. Sadidi et al. demonstrated that H_2_O_2_ activates PI3K and AKT and promotes survival of neuroblastoma SH-SY5Y cells [39]. This response was elicited by the PI3K/AKT-induced phosphorylation of proapoptotic Bax, which in turn suppresses apoptosis and promotes cell survival. An opposite effect was noted in GAB-expressing GBM cells, probably due to the lack of an active PI3K/AKT pathway which is functionally hampered by GAB expression. Accordingly, the addition of H_2_O_2_ to GAB-transfected cells does not allow further PI3K/AKT activation—as happens in GAB-silenced cells—and therefore, a decrease in cell survival and activation of apoptosis were seen in two GAB-transfected GBM cell lines. In addition, our previous study showed that overexpression of GAB in T98G GBM cells induced a strong downregulation of antiapoptotic Bcl-2 while proapoptotic Bid was overexpressed. Furthermore, overexpression of GLS2 decreased GBM cell survival, and this effect was increased by an oxidative insult (H_2_O_2_, arsenic trioxide) [22].

It has to be emphasized that our current results elucidate the mode of action of GAB in GBM cells exposed to oxidative stress. Further studies are required to establish whether GAB affects the PI3K/AKT pathway in GBM cells also in unstressed conditions.

In summary, we have shown that in the three cell lines examined so far, exogenous GAB decreases the survival and growth of GBM cells and sensitizes them to oxidative stress evoked by H_2_O_2_ treatment irrespective of their TP53/PTEN status. Moreover, the increased susceptibility of GAB-transfected cells to oxidative stress appears invariably related to the downregulation of the PI3K/AKT pathway. The study strongly favors the concept that the mechanism described above may universally hold for GBM cells of different origins regardless their genetic background and native tumorigenic potential.

## 4. Materials and Methods

### 4.1. Cell Culture and Transfection

T98G human GBM cell line (American Type Culture Collection, Manassas, VA, USA) was maintained in Earle’s Minimal Essential Medium (MEME) (Sigma-Aldrich, St. Louis, MO, USA) and supplemented with 10% fetal bovine serum (Gibco, Thermo Fisher Scientific, Grand Island, NY, USA), non-essential amino acids (Gibco), and 1% antibiotics (penicillin and streptomycin) (Gibco).

U87MG human GBM cell line (Sigma-Aldrich) was maintained in Eagle’s Minimum Essential Medium (EMEM) (ATCC, Manassas, VA, USA) and supplemented with 15% fetal bovine serum and 1% antibiotics (penicillin and streptomycin) (Gibco).

LN229 human GBM cell line (a kind gift from Rafał Krętowski, Department of Pharmaceutical Biochemistry, Medical University of Białystok, Białystok, Poland) was maintained in Dulbecco’s Modified Eagle Medium (DMEM) (Gibco) and supplemented with glucose (final concentration 4.5 g/L), 10% fetal bovine serum, and 1% antibiotics (penicillin and streptomycin) (Gibco).

All cell lines were maintained at 37 °C in a humidified atmosphere with 95% air and 5% CO_2_. T98G, U87MG, and LN229 cells were stably transfected with a pcDNA3 vector carrying a full cDNA sequence encoding human GAB or empty pcDNA3 vector, as described previously [21]. Transfection was performed using Lipofectamine2000 (Invitrogen, Grand Island, NY, USA) according to the manufacturer’s protocol. The culture medium for transfected cells (herein referred to as −GAB or −pcDNA) containing the neomycin-resistance gene was supplemented with 0.5 mg/mL G418 (BioShop, Lab Empire, Rzeszów, Poland) for T98G or U87MG transfectants or with 0.750 mg/mL G418 for LN229 transfectants. *GLS2* gene expression was monitored by RT-PCR.

All cell lines were authenticated by the profiling of short tandem repeats (STR) performed by ATCC and tested for mycoplasma contamination using Mycoplasma Detection Kit-Quick Test (Biotool, Stratech Scientific Limited, Cambridge, UK).

### 4.2. RNA Isolation and RT-PCR

Total RNA from the cells was extracted using TRI-Reagent (Sigma-Aldrich), according to the manufacturer’s protocol. The RNA concentration was measured using NanoDrop2000, and 2 µg of RNA were reverse-transcribed using a High Capacity cDNA Reverse Transcription Kit (Applied Biosystems, Warrington, UK) according to the manufacturer’s protocol. The cDNA fragments of GAB and β-actin (internal control) were amplified as described previously [40]. The PCR products were run on a 1% agarose gel and visualized using ethidium bromide dye.

### 4.3. Mitochondrial Activity Test (MTT)

The mitochondrial activity was determined by 3-(4,5-dimethylthiazol-2-yl)-2,5-diphenyl tetrazolium bromide (MTT) conversion into formazan. Briefly, 5 × 10^3^ cells per well were seeded in a 96-well plate and cultured for 48 h. After this time, the medium was removed and the cells were washed with phosphate-buffered saline (PBS) and incubated in the culture medium containing the MTT solution at the final concentration of 0.05 mg/mL for 1h to allow the conversion of MTT. Then the medium was replaced with dimethyl sulfoxide (DMSO), and the absorbance was read at 570 nm using an Elisa BioRad Microplate Reader. When the cells’ sensitivity to H_2_O_2_ was analyzed, 24 h after seeding, the cells were exposed to increasing concentrations of 5, 10, 25, and 50 µM of H_2_O_2_ for 15 and 30 min for the U87MG and LN229 cell lines and of 50, 100, 200, and 300 µM for 15 and 60 min for the T98G cell lines. After the treatments, the medium was removed, and the MTT test was performed. When the sensitivity of the cells to TMZ (Sigma-Aldrich) was investigated, 4 × 10^3^ cells per well were seeded in a 96-well plate and incubated in the culture medium for 24 h. After this time, the cells were exposed to increasing concentrations of 0, 10, 100, and 1000 µM of TMZ for 72 h. Then the culture medium was removed, and the mitochondrial activity was determined by the MTT test.

### 4.4. Proliferation Assay

The cells were seeded at the density of 5 × 10^3^ per well in a 96-well plate and cultured for 48 h. The cell proliferation was measured by bromodeoxyuridine (BrdU) incorporation using the Cell Proliferation Elisa BrdU (colorimetric) assay (Roche, Basel, Switzerland) according to the manufacturer’s instruction. The absorbance was read at 450 nm using an Elisa BioRad Microplate Reader. When the proliferation of the cells treated with TMZ was investigated, 4 × 10^3^ cells per well were seeded in a 96-well plate and cultured for 24 h. After this time, the cells were exposed to increasing concentrations of 0, 10, 100, and 1000 µM of TMZ for 72 h. Then, the culture medium was removed, and the BrdU assay was performed.

### 4.5. Colony Formation Assay

The cells were seeded at the density of 100 cells per well in a 6-well culture plate. After 3 weeks of culture, the medium was removed and the cells were fixed in 5% paraformaldehyde (PFA) for 1 h and then stained with 5% Giemsa solution. Readily visible colonies were counted.

### 4.6. Migration Scratch Assay

The migration was analyzed using a scratch assay. Monolayers of the cells were scratched by a tip and then incubated in the serum free culture medium. The scratch was photographed under a Juli Smart cell analyzer and measured at 0 and 24 h after scratching. Migration was determined by measuring the distance from the edges of the scratch.

### 4.7. Protein Isolation

Twenty-four hours after seeding, the cells were exposed to specified concentration of H_2_O_2_. After the incubation time, the cells were harvested and sonicated in a lysis buffer (50 mM Tris-HCl pH = 8.00, 150 mM NaCl, 5 mM EDTA, 0.5% NP-40) supplemented with cocktails of sodium fluoride, protease, and phosphatase inhibitors (Sigma-Aldrich). The lysates were centrifuged at 12,000× *g* for 10 min at 4 °C, and the supernatants were collected. The protein concentration was determined using the bicinchoninic acid Protein Assay Kit (Pierce, Rockford, IL, USA).

### 4.8. Western Blot

Thirty µg of proteins were separated on 10% SDS-PAGE and then electrotransferred to nitrocellulose membranes. The membranes were blocked with 5% bovine serum albumin (BSA) or 5% skim milk in TBST (20 mM Tris-HCl, pH 7.5, and 150 mM NaCl, containing 0.1% Tween 20) for 1 h at room temperature and then incubated with a primary antibody overnight at 4 °C. The following antibodies were used: p-AKT (Thr308) (#4056, Cell Signaling, Danvers, MA, USA), p-AKT (Ser473) (#4060, Cell Signaling), AKT (#4691, Cell Signaling), p-PDK1 (Ser241) (#ab109460, Abcam), PDK1 (#17086-1-AP, ProteinTech, Chicago, IL, USA), p-PI3K (Tyr199) (#4228, Cell signaling), PI3K (#11889, Cell Signaling), p-NF-κB (Ser536) (#MAB72261, Novus Biologicals, Littleton, CO, USA), and NF-κB (#4764, Cell Signaling) according to the manufacturer’s instruction. After washing in TBST, the membranes were incubated with a secondary anti-rabbit antibody (Sigma-Aldrich) conjugated to horseradish peroxidase for 1 h at room temperature. The blots were visualized on X-ray film using a SuperSignal West Pico Chemiluminiscence Substrate (Pierce). For loading control, the membranes were stripped two times for 15 min in a Stripping Buffer (0.1 M glycine, pH 2.9) and reused with an antibody against glyceraldehyde 3-phosphate dehydrogenase (#HRP-60004, ProteinTech). The densitometric analysis was performed using G:Box system and GeneTools software (Syngene, Frederick, MD, USA).

### 4.9. Real-Time PCR

Quantitative real-time PCR was performed by using 1 µL of cDNA mixed with TaqMan Fast Universal PCR Master Mix (Applied Biosystems) and the primers purchased from Applied Biosystems (AKT1, assay ID: Hs00178289_m1; PDPK1, assay ID: Hs00928927_m1; PIK3R3, assay ID: Hs01103591_m1) or Blirt (Gdańsk, Poland) (β-actin, cat no: HK-DD-hu). The reactions were incubated at 95 °C for 10 min, followed by 45 cycles of 95 °C for 3 s and 60 °C for 30 s using an Applied Biosystems 7500 Sequence Detection System. The relative expression was calculated using the ΔΔCT method [41] and normalized to the expression of β-actin.

### 4.10. Caspase Activity

The caspase activity was measured by using the Caspase-Glo 3/7 assay kit (Promega, Madison, Wisconsin, USA). Briefly, twenty-four hours after seeding, the pcDNA and GAB cells were treated with 200 µM (for T98G cell lines) or 5 µM (for U87MG and LN229 cell lines) H_2_O_2_ for 15 min. After this time, the caspase 3/7 reagent was added to the cells and incubated for 3 h at room temperature. The luminescence intensity was measured using a FLUOstar Omega (BMG Labtech, Ortenberg, Germany), and the raw data were presented as a percentage of control (pcDNA cells).

### 4.11. Statistical Analysis

Data were expressed as the mean ± SD from 3–7 independent experiments. The statistical analysis was performed using GraphPad Prism 5 (GraphPad Software, La Jolla, CA, USA). The statistical significance was determined by one-way analysis of variance (one-way ANOVA) followed by Tukey’s test (for multiple comparisons between more than two groups) or by Student’s *t*-test (for comparisons between two groups). *p* < 0.05 was considered as statistically significant.

## 5. Conclusions

**Figure 8:**
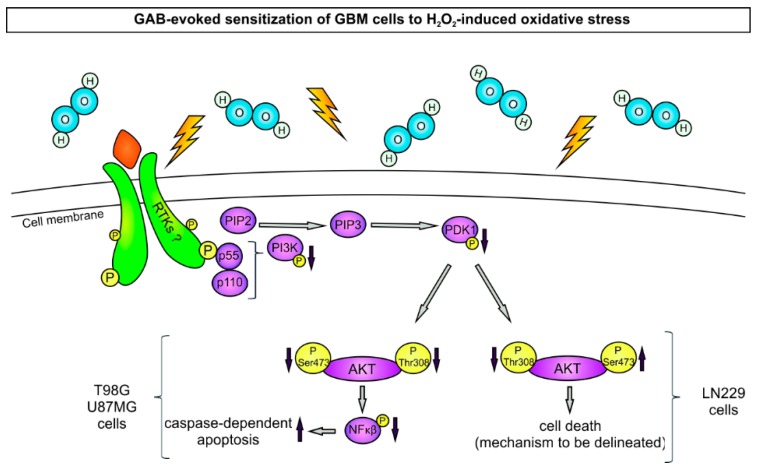
Transfection with GAB increases the sensitivity of GBM cell lines T98G, U87MG, and LN229 to H_2_O_2_-induced oxidative stress. Upon H_2_O_2_ treatment, the GAB-transfected cells of all three cell lines present decreased levels of phosphorylated PI3K and PDK1 compared to the pcDNA-transfected counterparts. Hypothetically, changes in the activity of the PI3K signaling cascade may ensue modulation of RTKs by GAB, but experimental evidence to support this notion is absent. GAB-transfected T98G and U87MG cells display decreased AKT phosphorylation on both Thr308 and Ser473, reduced NF-κB phosphorylation, and increased activity of caspase 3 and 7, suggesting that caspase dependent apoptosis contributes to their death. By contrast, in the LN229 cell line, the lack of changes in the phosphorylation level of NF-κB and in caspase 3 and 7 activities indicates that the mechanism underlying GAB-mediated death is other than caspase dependent apoptosis. Note that in contrast to GAB-transfected T98G and U87MG cells, GAB-transfected LN229 cells present increased AKT phosphorylation on Ser473: The implications (if any) of this difference for the nature of cell death are not known.

Our results show that GAB suppresses the malignant phenotype of GBM cells of different tumorigenic potentials and genetic backgrounds. Furthermore, transfection with GAB sensitizes GBM cells towards H_2_O_2_-mediated oxidative stress. Mechanistic studies revealed that the GAB-mediated increase of susceptibility to oxidative stress is causally related to the inhibition of the PI3K/AKT pathway (Figure 8). The upregulation of the GLS2 expression and the inhibition of the PI3K/AKT pathway may become a novel combined therapeutic strategy for anti-glioma preclinical investigations.

## Supplementary Materials

The following are available online at http://www.mdpi.com/2072-6694/11/1/115/s1, Figure S1: Analysis of GAB level in T98G cells wild-type (wt) or stably transfected with an empty pcDNA3 vector (pcDNA) or a pcDNA3 vector carrying GAB sequence (GAB), Figure S2: Transfection with GAB sequence reduces viability, proliferation, ability to form colonies, and ability to migrate T98G cells and enhances their sensitivity to H_2_O_2_.
