# Erratum: Majewska, E. et al. Transfection with GLS2 Glutaminase (GAB) Sensitizes Human Glioblastoma Cell Lines to Oxidative Stress by a Common Mechanism Involving Suppression of the PI3K/AKT Pathway. *Cancers* 2019, *11*, 115

**DOI:** 10.3390/cancers12082068

**Published:** 2020-07-27

**Authors:** Ewelina Majewska, Javier Márquez, Jan Albrecht, Monika Szeliga

**Affiliations:** 1 Department of Neurotoxicology, Mossakowski Medical Research Centre, Polish Academy of Sciences, 5 Pawińskiego Street, 02-106 Warsaw, Poland; emajewska@imdik.pan.pl (E.M.); jalbrecht@imdik.pan.pl (J.A.); 2 Canceromics Laboratory, Department of Molecular Biology and Biochemistry, Faculty of Sciences, Campus de Teatinos, Instituto de Investigación Biomédica de Málaga (IBIMA), University of Málaga, 29071 Málaga, Spain; marquez@uma.es

The authors wish to make the following corrections to this paper [1]: The information regarding one funding source was inadvertently indicated. The following acknowledgement of funding support should be removed: 

**Funding:** This research was funded by the National Science Centre of Poland grant number 2017/25/B/NZ7/00388 (to M.S.).

The correct funding is as below:

**Funding:** This research was funded by the National Science Centre of Poland grant numbers 2016/23/N/NZ5/01428 (to E.M.) and 2013/11/D/NZ7/00925 and by the National Leading Research Centre (KNOW-MMRC1) project (E.M.). J.M. was supported by Grant SAF2015-64501-R from the Spanish Ministry of Economy and Competitivity.

This change does not affect the scientific results. The manuscript will be updated and the original version will remain online on the article webpage, with a reference to this erratum. The authors would like to apologize for any inconvenience caused to the readers by this change. 
