# Supplementary material for: Transfection with GLS2 Glutaminase (GAB) Sensitizes Human Glioblastoma Cell Lines to Oxidative Stress by a Common Mechanism Involving Suppression of the PI3K/AKT Pathway
Source: Cancers (Basel). 2019 Jan 19;11(1):115. doi: 10.3390/cancers11010115 (PMC6356507; doi:10.3390/cancers11010115)
Supplement: Supplementary file 1 [file cancers-11-00115-s001.pdf]

cancers

Supplementary Materials: Transfection with GLS2 Glutaminase (GAB) Sensitizes Human Glioblastoma Cell Lines to Oxidative Stress by a Common Mechanism Involving Suppression of the PI3K/AKT Pathway

Ewelina Majewska, Javier Márquez, Jan Albrecht and Monika Szeliga

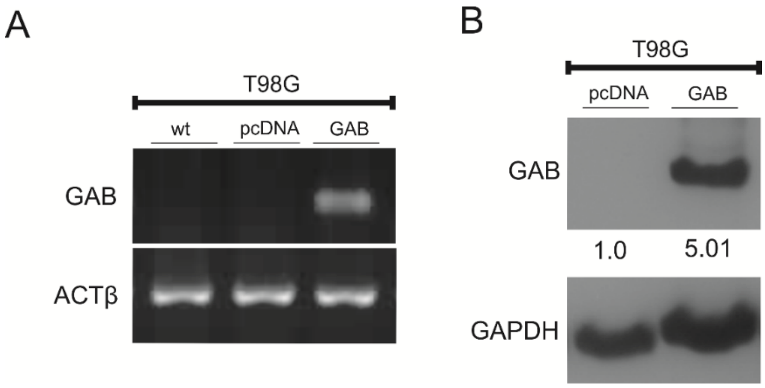

Figure S1. Analysis of GAB level in T98G cells wild-type (wt) or stably transfected with an empty pcDNA3 vector (pcDNA) or a pcDNA3 vector carrying GAB sequence (GAB). (**A**) GAB and ACTβ transcripts were determined by RT-PCR. (**B**) Protein levels of GAB and GAPDH in whole-cell lysates were determined by Western blot analysis. Rabbit anti-GLS2 antibody detecting both isoforms arising from GLS2 gene was used.

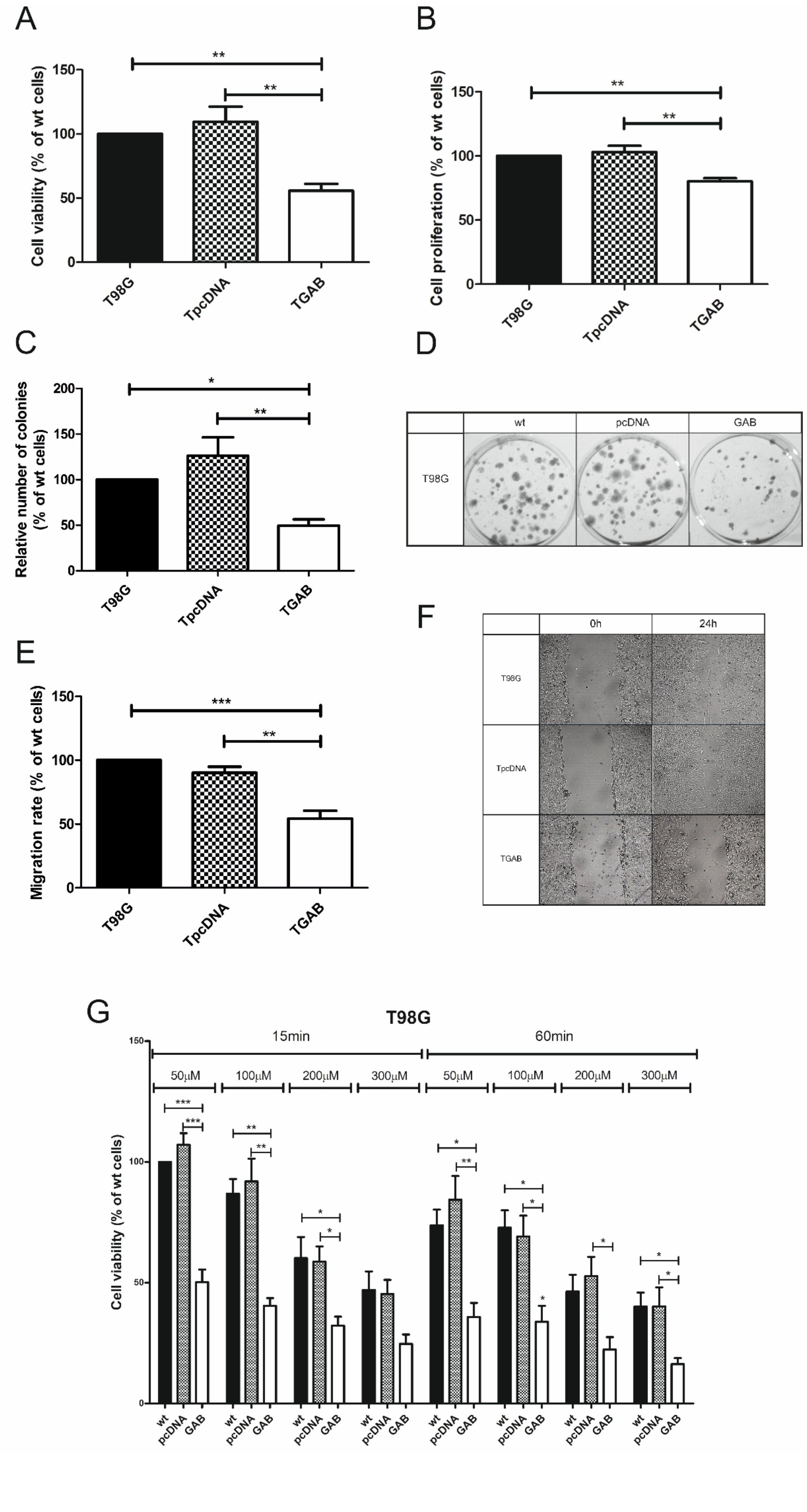
A
Cell viability (% of wt cells)
T98G
TpcDNA
TGAB
B
Cell proliferation (% of wt cells)
T98G
TpcDNA
TGAB
C
Relative number of colonies (% of wt cells)
T98G
TpcDNA
TGAB
D
wt
pcDNA
GAB
T98G
E
Migration rate (% of wt cells)
T98G
TpcDNA
TGAB
F
0h
24h
T98G
TpcDNA
TGAB
G
T98G
15min
60min
50μM
100μM
200μM
300μM
Cell viability (% of wt cells)
wt
pcDNA
GAB

Figure S2. Transfection with GAB sequence reduces viability, proliferation, ability to form colonies and ability to migrate of T98G cells and enhances their sensitivity to H_2O_2. (**A**) Mitochondrial activity of wild type (wt) cells or cells stably transfected with the indicated plasmids was assessed by the MTT test 48 h after seeding. Results are mean ± SD (n = 4) expressed as a percentage of wt cells. ** $p < 0.01$ versus wt and pcDNA cells (one-way ANOVA followed by Tukey's test). (**B**) Cell proliferation of wt cells or cells stably transfected with the indicated plasmids was assessed by the BrdU assay 48 h after seeding. Results are mean ± SD (n = 4) expressed as a percentage of wt cells. ** $p < 0.01$ versus wt and pcDNA (one-way ANOVA followed by Tukey's test). (**C**) Colony formation 3 weeks after seeding. Number of colonies are represented as a percentage relative to the number of colonies formed by wt cells. Results are mean ± SD (n = 4). * $p < 0.05$, ** $p < 0.01$ versus wt and pcDNA cells (one-way ANOVA followed by Tukey's test). (**D**) Representative images of the plates with colonies formed within 3 weeks of growth. (**E**) The migration rate of wt cells or cells stably transfected with the indicated plasmids was measured by the wound-healing. Twenty four hours after seeding, the confluent cells were scratch-wounded with a micropipette tip. Wound borders were recorded and measured at 0 and 24 h post-scratching. Results are mean ± SD (n = 3) expressed as a percentage of the scratch gap observed for wt cells. ** $p < 0.01$, *** $p < 0.001$ versus wt and pcDNA cells (one-way ANOVA followed by Tukey's test). (**F**) Representative images of scratch gaps taken at 0 and 24 h after scratching. Magnification : objective 4x and digital 10x. (**G**) Transfection with GAB sequence sensitizes T98G cells to treatment with H_2O_2. Viability of wt cells or transfected with the indicated plasmids was measured by the MTT test treatment with 50–300 μM H_2O_2 for 15 min and 1 h. Results are mean ± SD (n = 4) expressed as a percentage of wt cells treated with 5 μM H_2O_2 for 15 min. * $p < 0.05$, ** $p < 0.01$, *** $p < 0.001$ versus wt and pcDNA (one-way ANOVA followed by Tukey's test).

© 2019 by the authors. Licensee MDPI, Basel, Switzerland. This article is an open access article distributed under the terms and conditions of the Creative Commons Attribution (CC BY) license (http://creativecommons.org/licenses/by/4.0/).
